# From physical to social interactions: The relative entropy model

**DOI:** 10.1038/s41598-020-58549-8

**Published:** 2020-01-31

**Authors:** Yair Neuman, Dan Vilenchik, Yochai Cohen

**Affiliations:** 1 The Department of Cognitive and Brain Sciences and Zlotowski Center for Neuroscience, Ben-Gurion University of the Negev, Beer-Sheva, Israel; 2 School of Electrical and Computer Engineering, Ben-Gurion University of the Negev, Beer-Sheva, Israel; 3 Gilasio Coding, Tel-Aviv, Israel

**Keywords:** Information theory and computation, Applied mathematics

## Abstract

Complex social systems at various scales of analysis (e.g. dyads, families, tribes, etc.) are formed and maintained through verbal interactions. Therefore, the ability to (1) model these interactions and (2) to use models of interaction for identifying significant relations may be of interest to the social sciences. Adopting the perspective of social physics, we present a general approach for modeling interactions through relative entropy. For illustrating the benefits of the approach, we derive measures of “perspective-taking” and use them for identifying significant-romantic relations in a data set composed of the verbal interactions taken place at the famous TV series “Sex and the City”. Using these measures, we show that significant-romantic relations can be identified with success. These results provide preliminary support for the benefits of using the proposed approach.

## Introduction

Interactions are the hallmark of complex systems^1^ but in the social context, interactions are totally different from those evident among the particles of non-living matter^2^. This difference calls into question the general meaning of the term “interaction” and points to the need to better understand social interactions while still using physics as “point de repère”. In trying to address this challenge, one must be careful in bridging the gap between physics and the social sciences, as these two fields are not theoretically aligned and there is always the danger of using misleading analogies. In this context, one should also recall that in the social sciences the concept of interaction has been mostly used in a very general sense, covering a huge variety of instances from hugging to arguing. While there are some good reasons for this variety, it seems that it may be useful to use a general and physically motivated concept of interaction together with measures that are clearly derived from it. Adopting such an approach, may be relevant for the task of identifying *significant* relations/interactions. Not all social interactions are significant to the same extent and being able to identify significant interactions may be of interest both from a purely theoretical perspective and from a practical perspective (e.g. the development of interactive technologies such as Chatbots).

The aim of the current paper is to introduce a general conceptualization of interaction which is accompanied by operationalization through the use of relative entropy.

## Modeling Interactions Through Relative Entropy

We start by clarifying the idea of an interaction drawing on the approach proposed by Neuman *et al*.^3^ who study interactions through the perspective of statistical entropy and the concepts of additivity and non-additivity. Non-extensive systems, or “non-additive” systems are modeled using a non-additive form of entropy^4^, which is applied when the system that we analyse is characterized by strong correlations between its microstates and by emerging properties. Social systems are clearly non-additive as the system’s degrees of freedom are not proportional to the increase in the size of the system. For instance, when “merging” two interacting people into a collaborating dyad, the entropy of the merged dyad is non-additive, as described below. Neuman *et al*.^3^ describe non-additivity in a general conceptual sense as the nonlinear function associating the system’s granularity level of analysis and its components’ degrees of freedom. The way this idea is relevant for modeling interactions is explained through the next example.

When a couple interacts, they do not simply influence each other, as popular definitions of interaction may propose, but through their joint activity form a new social state (i.e. the dyad) with a unique and distinctive emerging signature. For instance, let’s take the trivial case of dating. Each person/particle involved in the date may have his own personal preferences for leisure activities and therefore for the best way for enjoying the date. However, as gender differences have been documented for leisure activities (e.g.^5–7^), we may hypothesize that the interaction between the men and the women participating in the date imposes mutual constraints that result in a joint dating activity that can be characterized by leisure activities probably different from each of the parties’ unique distribution of preferences or their simple sum. The entropy of a couple’s dating activities is therefore non-additive and different from the simple sum of each of the individuals’ preferences. The same idea may hold for the topics discussed during the date. Each participant in the date may be represented through the general discrete probability distribution of topics (e.g. sport, politics, art) characterizing his verbal interactions. However, the distribution of topics discussed during the specific date results from the mutual constraints imposed during the interaction and therefore may be quite different from each of the participants’ individual distributions. Following Neuman *et al*.^8^, we may therefore consider an interaction as involving mutual constraints through which a different scale of behavior/analysis emerges (e.g. the dyad’s behavior), having a distinctive pattern, which is different from the one of its particles and their simple composition.

Following the above example, we may think about each party who is involved in the interaction in terms of a distribution of behaviors (e.g. the distribution of leisure interests or topics for discussion), and about the interaction as the distribution describing their joint activity. For modeling an interaction, we may ask for instance, what is the extent to which the dyad’s distribution may be approximated using the distributions characterizing each of the participants in the interaction. This idea is general enough to cover various forms of interaction to include verbal interactions. For instance, in the series “Sex and the City” we have four main characters. When automatically analyzing the texts produced by each character, we may produce her distribution of topics. However, when a character like Carrie interacts with a romantic partner and a “significant other” such as “Mr. Big”, the distribution of their topical interests *as an interacting couple* may be quite different from the one characterizing each of them. This idea can be further explained using a different example of a significant relationship.

In the awarded film, “Good Will Hunting”, the hero Will Hunting, establishes deep relationships with his psychologist Dr. Maguire (played by Robin Williams). These relationships are evident in the topics they jointly discuss during the therapeutic sessions. We may describe the distribution of the topics generally characterizing Will Hunting as vector *G*. This is the distribution of topics that appears across his various social-verbal interactions, from those he maintains with his friends to those that he maintains with his girlfriend. Similarly, the distribution of topics generally discussed by Dr. Maguire may be tagged as *C*. The distribution characterizing the activity of Will and Dr. Maguire is the distribution of topics that they both discuss during the sessions and we may tag it as *GC*. These distributions may be used as the building blocks for modeling interactions and identifying significant relations. For example, one possible indication that the therapeutic sessions are significant to Will, at least from an information theoretic perspective, may be that there is a gap between Will’s general distribution of topics and the distribution produced during the therapeutic sessions. The “divergence” between these two distributions may be captured through the idea of relative entropy or Kullback-Leibler divergence, defined as follows:

$${D}_{KL}(P||Q)=\sum _{x}\,P(x)\log \,\frac{P(x)}{Q(x)}.$$

This measure gives us a clear indication of the “price” we should pay in bits of information when trying to code one distribution using another distribution. In the above example, we may use relative entropy to measure the “surprise” of observing Will’s interactions with Dr. Maguire given the a priori expectations formed by knowing Will’s usual behavior as represented by his general distribution of topic signals. The general conceptualization of interaction, which minimally involves a dyad (*G,C*), may be therefore modeled using the following distributions:

- *G* - the general discrete probability distribution of behaviors characterizing *G*
- *C* - the general discrete probability distribution of behaviors characterizing *C*
- *GC* - the joint discrete probability distribution characterizing the specific interactions of *G* and *C*

**Figure 1:**
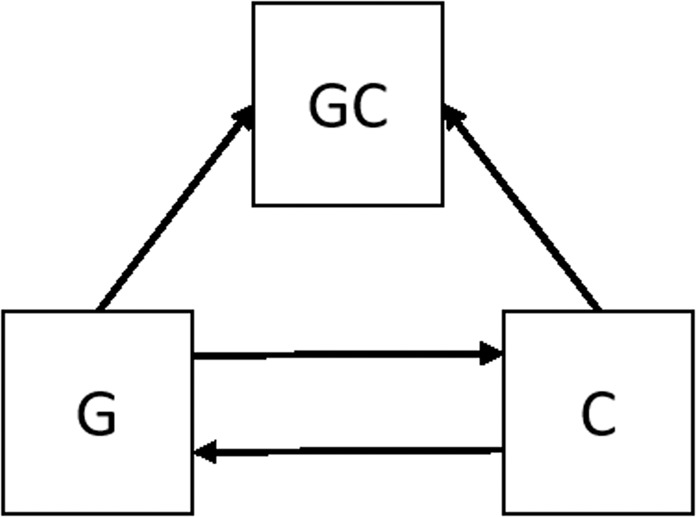
A schematic model of a dyadic interaction.

In our specific case, the distribution characterizing the behaviors of *G* will be a distribution over the topics that the character *G* discusses in its interactions with other characters. This distribution will be extracted using standard topic modeling tools (see description below). The general situation of interaction may be represented and analyzed through the diagram in Fig. 1, where an arrow from one distribution to the other, represents the relative entropy score of approximating one discrete probability distribution through the other. For instance, the higher is the relative entropy score *D*_*KL*_(*GC*||*C*), the more difficult it is to code the posterior distribution *GC* through the prior distribution *C*.

We ensure that all characters have the same distribution support. Hence, the variables used in the computation of *D*_*KL*_(*G*||*C*) for example, are the support of the distribution of *G* and *C*.

## Significant Interactions and Perspective-Taking

The relative entropy paths and their different compositions may serve as building blocks for building a model that may be used for successfully identifying significant relations. While the exact meaning of a “significant relation” may vary across domains, modeling such relations by using the relative entropy paths is general enough to cover a wide variety of cases. For instance, one may expect that a significant social interaction can be identified through the extent to which each party may “see” the joint activity he forms with a partner, through the other’s eyes. This ability is described in the psychological literature as “perspective-taking”^9^. Perspective-taking involves the ability to see the situation from the other’s point of view and it was found to be an important feature for establishing the common ground in a situated interaction^10^. Perspective-taking was also found to be highly important in the context of designing human-inspired robots, modeling human-robot interactions and social robotics^9,11–14^. The idea of ‘perspective taking’ corresponds with the idea discussed by Neuman *et al*.^3^ because perspective-taking involves the ability to mentalize a situation through the other’s perspective, hence through the constraints imposed by the other.

The idea of perspective taking may be explained through Fig. 1. Here we can see two different paths. The first from G to C to GC. The second from C to G to GC. Each arrow signifies the extent to which the co-domain distribution (e.g. GC) can be approximated by the domain distribution (e.g. C). The difference between the two paths (i.e. G, C, GC and C, G, GC) may serve as an indication to the extent to which *G* may “see” the joint activity GC through the constraints imposed by the other’s (i.e. C’s) own perspective and vice versa. Later, we define measures of perspective-taking that are based on this conceptualization (see the definition of *Persp*_*G*_ and *Persp*_*C*_ in the Evaluation section). If our proposed measures of perspective-taking validly represent significant interactions, then we should find that the perspective -taking scores may predict significant relations. We test this hypothesis as a first step in providing support for the benefits of using the proposed approach.

## Methodology

In the social sciences, it is extremely difficult to find a large data set of rich social-verbal interactions, which is accompanied by a clear criterion for tagging an interaction as “significant”. Therefore, for testing and validating the proposed approach and its derived measures, we have used a data set that meets these requirements.

This data set includes the transcribed verbal interactions of all of the characters that appear in the famous TV series “Sex and the City”. “Sex and the City” was a TV show that followed the lives of four women and their relations with “significant others” as well as with some other minor characters. The significant others are those characters having romantic relations with the main figures. As these significant others are clearly identified, the verbal interactions between them and the main characters may be used for testing our approach. We apply our conceptualization of interactions for analyzing this data set and for the task of identifying interactions with significant others. We show that these significant interactions can be identified with success. As such, we provide a first support for the practical benefits of using the proposed conceptualization and accompanied measures.

### The data

“Sex and the City” was a highly successful TV show that has been broadcasted for six seasons (1998 to 2004) and won numerous awards to include several Emmy awards. The series, which is set and located in New York City, describes the lives of four women: Carrie, Charlotte, Samantha and Miranda. During the seasons each of these characters is having relationships with what Wikipedia describes as “significant others”. Therefore, it is an appropriate data set for testing our ideas. For this purpose, we have used the Kaggle data set (available at https://www.kaggle.com/snapcrack/every-sex-and-the-city-script) that includes the scripts of all episodes and seasons, 283131 words and 37105 lines of text. The data set includes a running index, the name of the speaker (i.e. character) and the transcribed text produced by the speaker. Although this data set describes fictional human interactions, this fictional aspect doesn’t impede the relevance of the data set for testing our approach.

### Pre-processing

First, we have identified the significant interactions of Charlotte (with Trey and Harry), Carrie (with Mr. Big, Aidan, Jack and Aleksandr), Miranda (with Skipper, Robert, and Steve), and Samantha (with James, Maria, Richard and Smith). Although these interactions are evident to anyone who watched the series, we have used Wikipedia as a source for identifying the heroines’ significant relations. Next, for identifying a dialogue between two characters in the series, we have run for each episode a sliding window of length two and whenever a unique combination of characters appeared (e.g. Miranda and Skipper), extracted the text. We acknowledge the fact that this is only a limited heuristic for identifying interactions and as such consider it only as a practical solution for identifying the verbal interactions between two speakers given that there are no stylistic markers indicating where an interaction starts or ends. We removed interactions where one of the speakers produced less than 100 words throughout the season. Finally, we identified 418 interactions, out of which 13 are interactions with significant others, 311 are the interactions of the four main heroines with non-significant others, and the remaining 94 interactions do not include one of the four heroines. The latter interactions were removed from the data set.

Our next task was to compute the probability distributions of the topics for each participant and for the interactions of a given dyad. For the topic analysis, we have used Empath^15^, a software that performs automated topic analysis over a set of 194 pre-defined categories. Empath has been used in various contexts from suicide risk assessment^16^ to DARPA’S Low Resources Languages for the Emergent Incidents project (LORELEI)^17^, and up to the identification of personality disorders^18^.

We applied Empath in a straightforward manner to obtain, for example, the vector *G*, which is the distribution of topics produced by character *G* over all the interactions in which she has been involved (for brevity, we use the same notation for the character name and its associated vector). The vector *GC* is the distribution of topics produced by Empath when applied to the texts produced by both *G* and *C* during their mutual interactions and so on.

### Evaluation

In this section, we report the results of evaluating the various variables/features that were introduced so far for the Sex and the City data set. For testing our approach we have computed, for every interaction between characters *G* and *C*, the four variables described in Table 1. These variables are different measures of relative entropy representing different aspects of the interaction.

**Table 1:** The interaction features used in the analysis.

Feature	Description
*D*_*KL*_(*GC*||*G*)	The extent to which the topic distribution of the dyad *GC* is approximated by the distribution of the main character *G*
*D*_*KL*_(*GC*||*C*)	The extent to which the topic distribution of the dyad *GC* is approximated by the distribution of the side character *C*
*D*_*KL*_(*G*||*C*)	The extent to which the topic distribution of the main character *G* is approximated by the distribution of the side character *C*
*D*_*KL*_(*C*||*G*)	The extent to which the topic distribution of the side character *C* is approximated by the distribution of the main character *C*

In addition, we have formed three main variables of perspective-taking. To recall, our main hypothesis is that a significant interaction involves perspective-taking expressed in the ability to “see” the joint activity through the constraints imposed by the other party. To test this hypothesis, we propose three measures. The first two measures are presented below, where *G* always concerns one of the main characters and *C* its partner for the interaction:

$$\begin{array}{c}Pers{p}_{G}={D}_{KL}(GC||C)+{D}_{KL}(C||G)-{D}_{KL}(GC||G)\\ Pers{p}_{C}={D}_{KL}(GC||G)+{D}_{KL}(G||C)-{D}_{KL}(GC||C)\end{array}$$

A third measure of perspective-taking has been adopted from^19^ and represents the *redundancy* of the two parties in seeing their joint activity. It is defined as follows:

$$\rho (G,C)={|{D}_{KL}(GC||G)+{D}_{KL}(G||C)-({D}_{KL}(GC||C)+{D}_{KL}(C||G))|}^{-1}.$$

This measure aims to capture the extent to which the parties’ perspectives on the situation converge by factoring through each other.

To evaluate the predictive/classification power of each feature/variable *f*, we have applied the following general procedure. We have trained a single-variable logistic regression in which *f* is the independent variable, and *y*, the dependent variable, is the binary class indicator - significant or non-significant interaction. To remind the reader, in a logistic regression, one looks for the “best” linear relation between *f* and the odds of *y*:

1 $$\log (\tfrac{Pr[y\mathrm{=1}]}{Pr[y\mathrm{=0}])})=a\cdot f+b\mathrm{}.$$

A commonly used measure for the quality of the regression is the area under the ROC Curve, or simply AUC. The closer the AUC is to 1, the better is the classifier. The closer it is to 0.5, the more the classifier operates like a random coin toss. Since our classes are not balanced, we computed an average AUC using the following procedure. We repeated 20 times: sample 13 non-significant interactions and form a balanced data set of 26 interactions. Then we computed for this data set the AUC of a 3-fold cross validation. Finally, we have averaged the results over the AUC of the 20 executions.

### Controlling the conversation’s length

**Figure 2:**
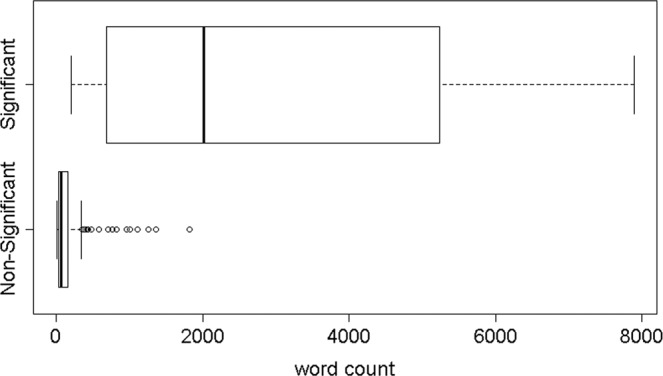
Word count distribution for significant vs. non-significant interactions across all seasons.

A natural and quite trivial feature which is indicative of the significance level of the interaction is its length. We tend to interact more intensively with people who are significant to us (e.g. spouse) and more briefly with non-significant others (e.g. the cashier at the gas station). The box-plot in Fig. 2 shows the distribution of the interaction lengths of the two classes: the interactions of the main four characters with their significant others (13 interactions in total), and their interactions with non-significant others (311 in total). As clearly seen from the box-plot, the length of the interaction is highly indicative of the type of the interaction (significant or not). Indeed, the AUC of a logistic-regression trained with the *total_number_of_words* feature produced an AUC of 0.98.

The aim of this paper is mainly to test the predictive value of our interaction-based features regardless of the interaction’s length or the predictive power of other features. It must be emphasized that in the current paper, we have no interest in gaining the best classification rate of significant interactions using the textual signature of the interaction. In such a practical case, we would have applied advanced tools of Machine Learning (e.g. XGBoost) and would have used all possible features, including the length of the interaction. In other words, the justification for our information-based approach is mainly theoretical, rather than an attempt to gain the best performance in identifying significant interactions.

Nevertheless, the length of the interaction, which is clearly an important cue for identifying significance, might inject noise into our analysis. The reason is that when running a topic analysis tool (e.g. Empath) on a larger text, the relative entropy measures may show that we can better code one distribution through the other simply because their mutual support is larger. Put differently, when computing the *D*_*KL*_ distance to a sparser distribution, we expect the distance to be larger as the mutual support will typically be smaller.

**Figure 3:**
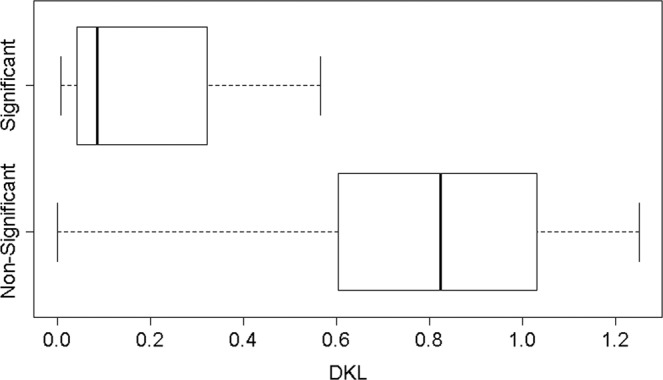
*D*_*KL*_(*GC*||*G*) feature distribution for significant vs. non-significant interactions across all seasons.

Figure 3 presents the distribution of the variable *D*_*KL*_(*GC*||*G*), which is the *D*_*KL*_ score measuring the divergence between the heroine interaction with another character and the heroine general distribution of topics. Indeed the box-plot corroborates our intuition. Correspondingly, the AUC of both *D*_*KL*_(*GC*||*G*) and *D*_*KL*_(*GC*||*C*) was found to be very high, 0.92 and 0.89 respectively.

**Figure 4:**
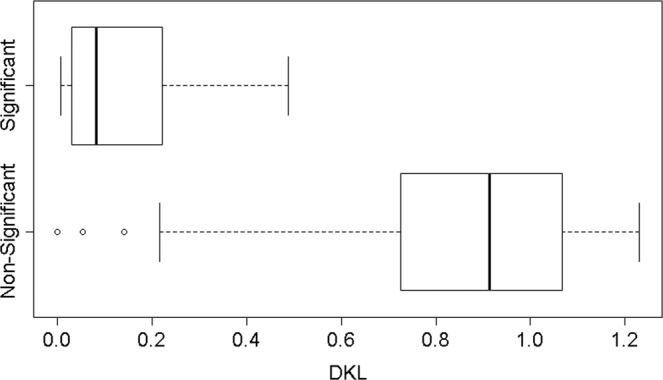
*D*_*KL*_(*GC*||*G*) feature distribution for significant vs. non-significant interactions with Wikipedia text instead of real text.

To exemplify how indeed the differences depicted in Fig. 3 may simply be an artifact of the conversation’s length, we conducted the following experiment. In the raw data file (that contains the dialogues of the entire series), we replaced the text of each character with a random text of the same length that has been retrieved from Wikipedia. We repeated the entire computation that produced the Empath vectors, and compared the new values of *D*_*KL*_(*GC*||*G*) across the two classes of interaction. As evident from Fig. 4, the distributions are still separated in roughly the same manner and the AUC of *D*_*KL*_(*GC*||*G*) is 0.94.

Therefore, we concluded that in order to validly test the predictive power of our model and measures, we must control the confounding variable of the interaction’s length as evident in the number of words composing it. To balance the length of the significant and non-significant interactions, we added the following pre-processing step.

For each main character, let ${S}_{1},\ldots ,{S}_{k}$ be its significant-others and ${N}_{1},\ldots ,{N}_{r}$ its non-significant others. We merged ${N}_{1},\ldots ,{N}_{i}$ into a new character ${N}_{1}^{\ast }$, where *i* is chosen such that the total length of the interaction with ${N}_{1},\ldots ,{N}_{i}$ is roughly the same length of the interaction with *S*_1_. This way we continued merging characters, ending up with a new set of 27 non-significant others ${N}_{1}^{\ast },\,\ldots ,\,{N}_{27}^{\ast }$, with the desired property that the length distribution is now roughly the same in the two classes. Using this procedure, we have formed an “experiment” group corresponding with the class of significant interactions and a matched “control” group corresponding with the class of non-significant interactions.

**Figure 5:**
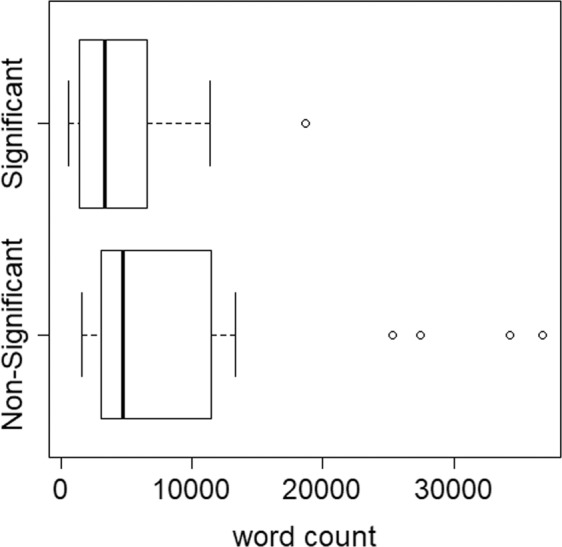
Word count distribution for significant vs. non-significant interactions across all seasons after merging characters.

Figure 5 shows the new length distribution of significant vs. non-significant interactions, which is much more balanced, a fact which is also reflected by the lower value of the AUC (AUC = 0.55). The analyses that follow all refer to the new data set with its balanced experiment and matched control groups.

### A non-parametric approach

First, we have used a non-parametric test for examining whether there is a statistically significant difference between the experiment and control groups. We used the Mann-Whitney U Test with a Monte Carlo simulation of 10,000 samples and a 99% CI. For the interaction measures, the difference was found to be significant (*p* < 0.001) for *D*_*KL*_(*G*||*C*) and *D*_*KL*_(*C*||*G*) (*z* = 4.10 and *z* = 4.12 respectively). For the perspective-taking measures the difference was found to be statistically significant (*p* < 0.001) for *persp*_*G*_ and *persp*_*C*_ and for the redundancy measure *ρ*(*G,C*) (*z* = 3.83, z = 4.17 and *z* = 3.32 respectively).

### A parametric approach

We have tested the predictive power of the variables (in the limited sense of “predictive” as curve fitting). Table 2 describes the performance of the logistic regression analysis.

**Table 2:** Performance of logistic regression for Interaction’s Significance. The reported results are the average of 20 executions, each with 3-fold cross validation. Using ${\chi }^{2}$-test of the residual deviance,

Feature	AUC
*D*_*KL*_(*GC*||*G*)	0.91
*D*_*KL*_(*GC*||*C*)	0.92
*D*_*KL*_(*C*||*G*)	0.93
*D*_*KL*_(*G*||*C*)	0.92
*Presp*_*G*_	0.92
*Presp*_*C*_	0.92
$\rho (G,C)$	0.83

all regressions were found to be statistically significant.

As evident from the table, even when controlling the length of the interaction, the perspective-taking variables are found to be statistically significant predictors of the relations’ significance. We repeated the experiment with a Linear Discriminant Analysis (LDA) classifier instead of logistic regression. The results were very similar.

The regression coefficient *a* (see Eq.(1) for the definition of *a*) was positive in all the logistic regressions. It means, that according to the statistical model, the larger the value of the variable, the higher the chances of a significant relation. It must be emphasized, that we did not produce any “one-tailed” hypothesis regarding the sign of the coefficient and the reason is that in some contexts of interaction it may be that the lower the relative entropy score the higher the chances are that the interaction is significant (negative coefficient). In other cases, the direction may be opposite (positive coefficient) as in the context of our romantic interactions. The sign of the coefficient in the above-mentioned results can be attributed to the fact that the topics discussed by the interacting romantic partners may be quite different from the topics each of them discusses in other contexts and in less significant interactions. As our approach is generic, it provides the measures regardless of the exact signs of the coefficients in the model.

## Discussion

Modeling social processes through the perspective of physics is not a trivial task partially as both theoretical and methodological bridges must be constructed between the abstract ideas and the concrete measures. In this paper, we have followed the proposal of Neuman *et al*.^3^ and the idea of social systems as non-additive systems. Drawing on this idea, we have proposed to measure perspective-taking drawing on the relative-entropy measure and used it for identifying significant-interactions in verbal-textual data. It is clear, that interactions can take various forms. In the context of soccer games they can take the form of ball passes leading to scoring a goal, in the context of romantic interactions they can take the form of flirting and so on. However, in both biological and social system an interaction involves spatially and temporarily reciprocal actions in which information/matter is exchanged under certain constraints for functional aims. Through mutual constraints, a higher form of organization is than constructed (e.g. a couple) in a way that restricts the degrees of freedom associated with each isolated particle and in a way that provides a lawful and functionally-distinct behavior at the macro-state level (e.g. the dyad). This general logic is evident in the behavior of various systems from the immune system^20^ to human language^21^ and usually involves an emerging behavior clearly identified by the social sciences and psychology. Here, we provide a simple and general way for modeling verbal interactions along the lines described above. While the proposed approach might be portrayed as too simple for analysing the complexity of social interactions, it can be trivially extended to social interactions involving more than two people and to many other small systems that are of interest to the social sciences^22^. It is not clear though, how well the proposed approach scales up to the level of populations.

In sum, in this paper we have proposed a general approach for the modeling of interactions and provided preliminary support for the potential benefits of using it. Our study is limited both in scope and size. Nevertheless, the minimal support that we have provided, may encourage future research into the way interactions constitute the formation of various social systems.

## Data Availability

The dataset used during the current study is available at, https://www.kaggle.com/snapcrack/every-sex-and-the-city-script.
